# Long-term effects of short-term postoperative bromfenac on macular thickness after phacovitrectomy for idiopathic epiretinal membrane

**DOI:** 10.1038/s41598-026-50272-0

**Published:** 2026-06-06

**Authors:** No Hae Park, Bo Hyun Park, Ik Soo Byon, Sungwho Park

**Affiliations:** 1 Department of Ophthalmology, Pusan National University School of Medicine, Pusan, Republic of Korea; 2 Baekrok Eye Clinic, Pusan, Republic of Korea; 3 Department of Ophthalmology, National University Hospital Biomedical Research Institute, 179, Gudeok-Ro, Seo-Gu, Busan, 49241 Republic of Korea

**Keywords:** Bromfenac, Cystoid macular edema, Epiretinal membrane, Macular thickness, NSAID, Diseases, Medical research

## Abstract

Postoperative macular edema is a recognized complication following vitrectomy for idiopathic epiretinal membrane (ERM), particularly after combined phacovitrectomy. Topical nonsteroidal anti-inflammatory drugs (NSAIDs) inhibit prostaglandin-mediated disruption of the blood-retinal barrier, but their long-term anatomical impact after ERM surgery has not been established. We retrospectively reviewed 282 eyes from 279 patients who underwent vitrectomy or phacovitrectomy for idiopathic ERM. Patients were categorized into an Early Use Group (topical bromfenac initiated within 4 weeks postoperatively, n = 91) and a Late/No Use Group (n = 191). The primary outcome was the change in central subfield macular thickness (CSMT) measured by OCT over 6 months. Longitudinal analysis was performed using a linear mixed-effects model (LME) with treatment-by-time interaction as the primary term. LME analysis revealed a significant treatment-by-time interaction for CSMT in the full cohort (F = 4.62, *p* = 0.003) and in the phacovitrectomy subgroup (F = 6.23, *p* < 0.001). The Early Use Group showed a significantly greater reduction in CSMT at 6 months (93.9 ± 56.2 μm) compared with the Late/No Use Group (76.6 ± 52.8 μm), with an estimated marginal mean difference of approximately 14–15 μm maintained at all post-baseline time points (*p* ≤ 0.006). For logMAR BCVA, a significant treatment-by-time interaction was also observed (F = 2.68, *p* = 0.046): the Early Use Group showed significantly faster visual recovery at 1 month (*p* = 0.016), but both groups converged to equivalent visual acuity by 6 months (*p* = 0.305). Early postoperative bromfenac within 4 weeks of phacovitrectomy for ERM significantly reduces macular thickness, and this anatomical benefit is durably maintained for at least 6 months, along with accelerated visual acuity recovery at 1 month. These findings raise the hypothesis that prostaglandin-mediated vascular permeability in the early postoperative period may influence long-term macular remodeling; however, this remains speculative, as direct evidence of permeability changes was not assessed in this study.

## Introduction

Idiopathic epiretinal membrane (ERM) is an avascular fibrocellular membrane proliferation between the vitreous and internal limiting membrane (ILM) over the macular area^1^. The standard treatment for ERM is vitrectomy with ERM removal, with or without ILM peeling^2,3^. Even after membrane removal, irreversible visual impairments may occur, caused by not only mechanical traction but also disruption of the blood-retinal barrier^4–6^. Postoperative macular edema occurs in approximately 10–15% after ERM removal, and prostaglandins seem to attribute to it^7^.

ERM exerts mechanical traction on the inner retinal surface, progressively thickening inner retinal layers including the ganglion cell complex (GCC) and retinal nerve fiber layer (RNFL)^8,9^. Following surgical ERM removal, relief of this traction leads to progressive thinning of these inner retinal layers, which correlates with visual acuity improvement^8,9^. This establishes that the natural biological trajectory after ERM surgery is one of progressive inner retinal thinning, not thickening.

Topical nonsteroidal anti-inflammatory drugs (NSAIDs), which inhibit prostaglandins, reduce the incidence of pseudophakic cystoid macular edema (CME)^10^ after cataract surgery and are superior to topical steroids in the management of pseudophakic CME.

Previous studies have analyzed the impact of topical NSAIDs on macular thickness and morphology after vitreoretinal surgery; however, the results were contradictory^11–13^. Over the past decade, there have been many changes in ERM surgery, including ILM peeling and combined cataract surgery, which are well known to affect macular thickness^7,14,15^. Therefore, the impact of topical NSAIDs should be reevaluated in the context of current surgical practices.

In our previous study^16^ analyzing the incidence of pseudophakic CME after phacovitrectomy for ERM by a single surgeon, short-term use of postoperative bromfenac prevented pseudophakic CME on postoperative one month. In the present study, we aimed to determine whether the short-term use of postoperative bromfenac has a long-term effect on postoperative macular thickness changes after phacovitrectomy for ERM by a multi-surgeon setting.

## Methods

We retrospectively reviewed the medical records of consecutive patients diagnosed with idiopathic ERM who underwent vitrectomy or phacovitrectomy for ERM removal at Pusan National University Hospital (Pusan, Korea) and Pusan National University Yangsan Hospital (Yangsan, Korea) between January 2015 and July 2022. The current study was approved by the Institutional Review Board of Pusan National University Hospital (2401–025-135) and was conducted in accordance with the principles of the Declaration of Helsinki. Informed consent was obtained from all subjects.

Idiopathic ERM was confirmed by the presence of a fibrous membrane on the macula via fundus photography and swept-source OCT (SS-OCT, Topcon DRI OCT-1 Atlantis; Topcon Inc, Tokyo, Japan). ERM was structurally graded into stages 1 through 4 according to the OCT-based staging system proposed by Govetto et al.^17^. We excluded ERM secondary to retinal vascular diseases such as diabetic retinopathy and retinal vein occlusion, uveitis, age-related macular degeneration, high myopia (spherical equivalent ≥ -5.0 diopters), and retinal breaks with retinal detachment. We also excluded eyes showing features of lamellar holes and pseudoholes due to limitations in the accuracy of macular thickness measurement.

All phakic eyes underwent phacovitrectomy, whereas pseudophakic eyes underwent vitrectomy alone. In cases of phacovitrectomy, eyes where an intraocular lens (IOL) was inserted out-of-the-bag due to posterior capsule rupture or zonule dialysis were excluded. Patients were classified into two groups: the Early Use Group, in which topical bromfenac was administered within 4 weeks after surgery, and the Late/No Use Group, in which topical bromfenac was initiated after postoperative week 4 or was not used. As this was a retrospective study involving five or more surgeons, no standardized protocol governed the timing of bromfenac initiation. In the Late/No Use Group, bromfenac was generally initiated when CME was suspected, visual acuity improvement was deemed insufficient, or the patient reported persistent ocular discomfort. The absence of a uniform criterion for late initiation reflects the real-world, multi-surgeon nature of this study and represents an inherent limitation of its retrospective design.

The primary outcome was the change in central subfield macular thickness (CSMT) measured by OCT between the two groups. The secondary outcome was best-corrected visual acuity (BCVA). Additionally, we analyzed differences between phacovitrectomy and vitrectomy subgroups.

Surgical Procedures. Retrobulbar anesthesia was administered before surgery. Pars plana vitrectomy was performed using a sutureless 25-gauge vitrectomy system (Constellation; Alcon Laboratories, Inc., Fort Worth, TX, USA) and a non-contact viewing system (Resight 700; Carl Zeiss Meditec AG, Jena, Germany). The ERM and ILM were peeled circularly and tangentially to the retinal surface using end-gripping intraocular forceps under a biconcave contact lens. The ILM was stained using Brilliant Blue G (0.025 mg/ml). In all phacovitrectomy cases, phacoemulsification and IOL implantation were performed prior to vitrectomy.

Postoperative Management. Postoperatively, all patients received topical 0.5% moxifloxacin (Moroxacin; Alcon) and topical 1% dexamethasone (Maxidex; Alcon) eye drops four times daily for the first week. At the postoperative week one visit, depending on the surgeon’s discretion, topical antibiotics and steroids were either continued or discontinued. Topical 0.1% bromfenac (Bronuck, Taejun, Seoul, Korea) could be initiated or stopped at any postoperative time point according to the surgeon’s discretion.

Ocular Examination. Patients underwent comprehensive ophthalmological examinations, including BCVA measurements, indirect ophthalmoscopy, fundus photography, and OCT at baseline and at 1, 3, and 6 months postoperatively. BCVA was measured using the Snellen chart and converted to the logarithm of the minimum angle of resolution (logMAR) scale for statistical analysis. CSMT was defined as the thickness of the innermost 1 mm ring on the ETDRS grid in the radial scan provided by the OCT system.

Statistical Analysis. Baseline characteristics between the two groups were compared using the Mann–Whitney U test and chi-squared test as appropriate. To evaluate the longitudinal effect of bromfenac on CSMT and BCVA over time, a linear mixed-effects model (LME) was employed with time (baseline, 1, 3, and 6 months) and group (Early Use vs. Late/No Use) as fixed effects, patient as a random effect, and age, sex, surgery type, and baseline CSMT as covariates. The treatment-by-time interaction term (time × group) was the primary term of interest, as it directly tests whether the two groups followed different trajectories of macular thickness change. Estimated marginal means (EMMs) were derived at each time point to quantify between-group differences while adjusting for covariates. LME analyses were performed using R software (version 4.x; R Foundation for Statistical Computing, Vienna, Austria) with the lme4, lmerTest, and emmeans packages. Changes in BCVA and CSMT between baseline and follow-up visits were additionally analyzed using the Wilcoxon signed-rank test. The impact of the duration and initial timing of bromfenac use was assessed using Spearman’s rank correlation. All other analyses were performed using SPSS software (version 22.0; IBM Corp., Armonk, NY, USA). A *P*-value of < 0.05 was considered statistically significant.

## Results

A total of 282 eyes from 279 patients were enrolled in the study. Ninety-one eyes (32.2%) were included in the Early Use Group, and the remaining 191 eyes (67.8%) were included in the Late/No Use Group.

*Baseline Characteristics:* There were no significant differences in baseline characteristics between the two groups, including age (*P* = 0.261), sex (*P* = 0.580), proportion of phacovitrectomy (*P* = 0.352), preoperative visual acuity (*P* = 0.206), preoperative CSMT (*P* = 0.149), or ERM stage distribution (*P* = 0.832) (Table 1). ERM stages were distributed as follows: in the Early Use Group, stage 1 in 9 eyes (9.8%), stage 2 in 31 eyes (34.0%), stage 3 in 37 eyes (40.6%), and stage 4 in 14 eyes (15.6%); in the Late/No Use Group, stage 1 in 23 eyes (12.0%), stage 2 in 71 eyes (37.2%), stage 3 in 73 eyes (38.2%), and stage 4 in 24 eyes (12.6%). In the Early Use Group, patients applied bromfenac for an average of 12.6 ± 6.3 weeks. In the Late/No Use Group, 27 (14.1%) of 191 eyes also received bromfenac after postoperative week 4 for an average duration of 10.2 ± 5.1 weeks.

**Table 1:** Baseline characteristics.

	Early Use Group	Late/No Use Group	*p-*value
Number of eyes, n	91	191	
Male / Female, n	35 / 56	67 / 124	0.580
Age (years)	63.0 ± 7.0	66.4 ± 7.3	0.261
Phacovitrectomy / Vitrectomy, n	78 / 13	171 / 20	0.352
Baseline CSMT (um)	445.3 ± 71.5	432.4 ± 69.3	0.149
Baseline BCVA (logMAR)	0.36 ± 0.22	0.39 ± 0.25	0.206
ERM stage, n			0.832
Stage 1	9 (9.8%)	23 (12.0%)	
Stage 2	31 (34.0%)	71 (37.2%)	
Stage 3	37 (40.6%)	73 (38.2%)	
Stage 4	14 (15.6%)	24 (12.6%)	

Data is presented as the mean ± SD^a^ or number of patients.CSMT, central subfield macular thickness. BCVA, best corrected visual acuity. *P < 0.05 statistically significant.

*Macular Thickness Changes* (Table 2): The baseline mean CSMT was 445.3 ± 71.5 μm in the Early Use Group and 432.4 ± 69.3 μm in the Late/No Use Group. During the first 4 postoperative weeks, the reduction of CSMT was 60.9 ± 50.6 μm in the Early Use Group, which was significantly higher than the 44.9 ± 50.4 μm reduction observed in the Late/No Use Group (*P* = 0.012).

**Table 2:** Central subfield macular thickness (CSMT, μm) by group and time point.

Time point	Early Use Group (n = 91)	Late/No Use Group (n = 191)	*p*-value
Baseline	445.4 ± 72.0	432.4 ± 69.5	0.135
1 month	384.5 ± 54.8	387.8 ± 55.1	0.574
3 months	363.1 ± 47.0	366.2 ± 45.1	0.621
6 months	351.5 ± 42.5	355.9 ± 45.8	0.377

Data are mean ± SD. p-values from Mann–Whitney U test. LME analysis: Time × Group interaction F = 4.62, *p* = 0.003 (full cohort). CSMT, central subfield macular thickness.

**Fig. 1:**
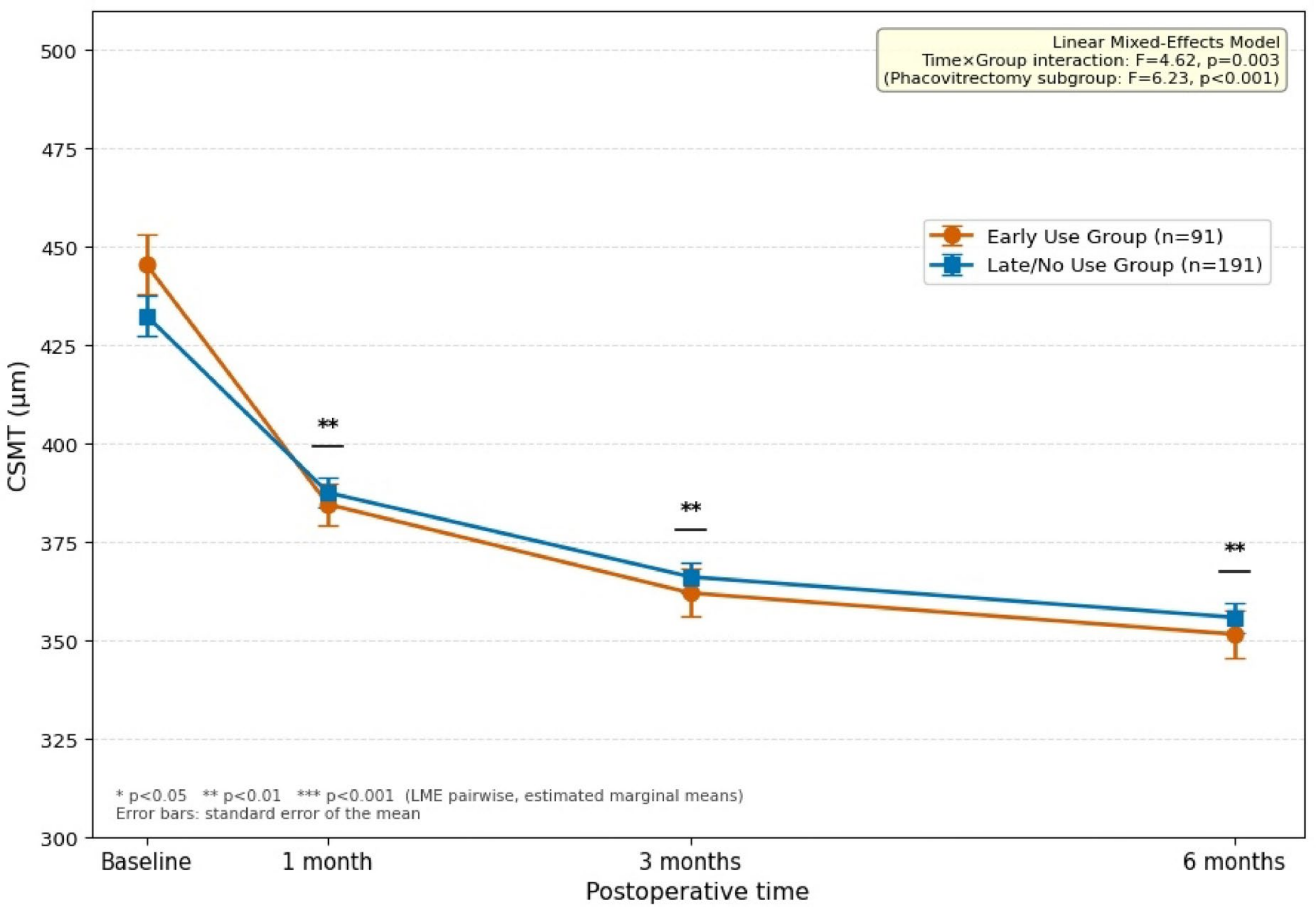
Changes in Central Subfield Macular Thickness (CSMT). Linear mixed-effects model (LME) revealed a significant treatment-by-time interaction for CSMT in the full cohort (F = 4.62, *P* = 0.003) and in the phacovitrectomy subgroup (F = 6.23, *p* < 0.001). Estimated marginal mean (EMM) comparisons showed no significant between-group difference at baseline (*P* = 0.164), but the Late/No Use Group had significantly greater CSMT at 1 month (*P* = 0.006), 3 months (*P* = 0.004), and 6 months (*P* = 0.003), indicating that early bromfenac use established an anatomical advantage within the first postoperative month that was durably maintained throughout the 6-month follow-up. Significance markers indicate LME pairwise comparisons of estimated marginal means. Error bars represent standard error of the mean. Orange circles, Early Use Group; Blue squares, Late/No Use Group.

From postoperative week 4 to week 12, there was no significant difference in CSMT reduction between the two groups (22.2 ± 29.5 μm in the Early Use Group vs. 21.8 ± 32.7 μm in the Late/No Use Group, *P* = 0.506). Similarly, from postoperative week 12 to week 25, no significant difference was found (11.1 ± 19.1 μm vs. 10.1 ± 21.8 μm, *P* = 0.857) (Fig. 1). However, due to the initial rapid reduction, the total CSMT reduction at 6 months after surgery was significantly greater in the Early Use Group (93.9 ± 56.2 μm) compared to the Late/No Use Group (76.5 ± 52.6 μm) (*P* = 0.012).

**Fig. 2:**
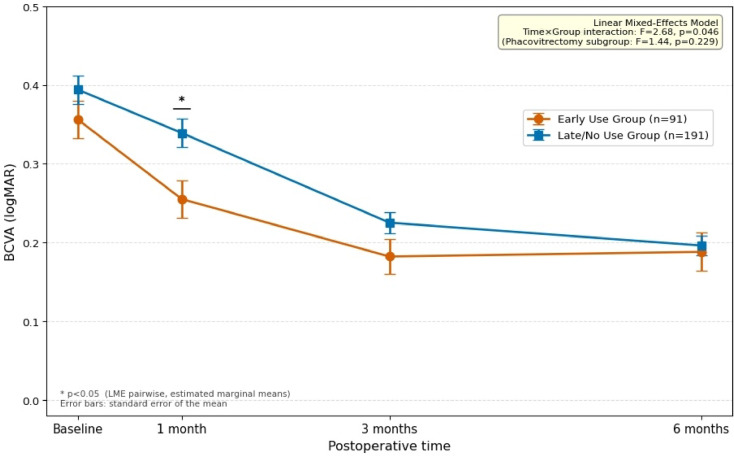
Changes in Best-Corrected Visual Acuity (BCVA, logMAR). LME analysis revealed a significant treatment-by-time interaction for logMAR BCVA in the full cohort (F = 2.68, *P* = 0.046). The Early Use Group showed significantly faster visual recovery at 1 month (EMM: 0.280 vs. 0.333 logMAR, *P* = 0.016), whereas no significant difference was observed at 3 months (*P* = 0.503) or 6 months (*P* = 0.305), indicating convergence of visual outcomes. Note that lower logMAR values indicate better visual acuity. Significance markers indicate LME pairwise comparisons of estimated marginal means. Error bars represent standard error of the mean. Orange circles, Early Use Group; Blue squares, Late/No Use Group.

*Visual Acuity Outcomes* (Table 3)*:* The baseline mean best-corrected visual acuity (BCVA, logMAR) was 0.36 ± 0.22 in the Early Use Group and 0.39 ± 0.25 in the Late/No Use Group, with no significant difference at baseline (EMM: Late/No Use 0.389 vs. Early Use 0.381, *P* = 0.716) (Fig. 2). LME analysis revealed a significant treatment-by-time interaction for logMAR BCVA in the full cohort (F = 2.68, *P* = 0.046), indicating that the two groups followed different trajectories of visual recovery over the follow-up period. At 1 month, the Early Use Group demonstrated significantly better visual acuity than the Late/No Use Group (EMM: 0.280 vs. 0.333, Δ =  − 0.054 logMAR, *P* = 0.016), reflecting accelerated early recovery. At 3 months, the difference was no longer significant (EMM: 0.205 vs. 0.220, *P* = 0.503). By 6 months, both groups showed similar visual acuity with no significant difference (EMM: 0.213 vs. 0.191, *P* = 0.305), indicating convergence of visual outcomes. In the phacovitrectomy subgroup, the treatment-by-time interaction was not significant (F = 1.44, *P* = 0.229), though a significant Early Use advantage was observed at 1 month (*P* = 0.026) with no significant differences at subsequent time points. In the vitrectomy-only subgroup, no significant interaction was observed (F = 2.18, *P* = 0.096), consistent with the CSMT findings.

**Table 3:** Best-corrected visual acuity (BCVA, logMAR) by group and time point.

Time point	Early Use Group (n = 91)	Late/No Use Group (n = 191)	*p*-value
Baseline	0.356 ± 0.226	0.394 ± 0.247	0.297
1 month	0.255 ± 0.228	0.339 ± 0.249	0.005*
3 months	0.182 ± 0.207	0.225 ± 0.188	0.011*
6 months	0.188 ± 0.230	0.196 ± 0.177	0.105

Data are mean ± SD. p-values from Mann–Whitney U test (between groups at each time point); *p < 0.05. Note: LME-based EMM pairwise comparisons yield different p-values (see Results text), as the LME model adjusts for covariates and within-subject correlation. LME analysis: Time × Group interaction F = 2.68, *p* = 0.046 (full cohort). Lower logMAR = better visual acuity. BCVA, best-corrected visual acuity.

**Fig. 3:**
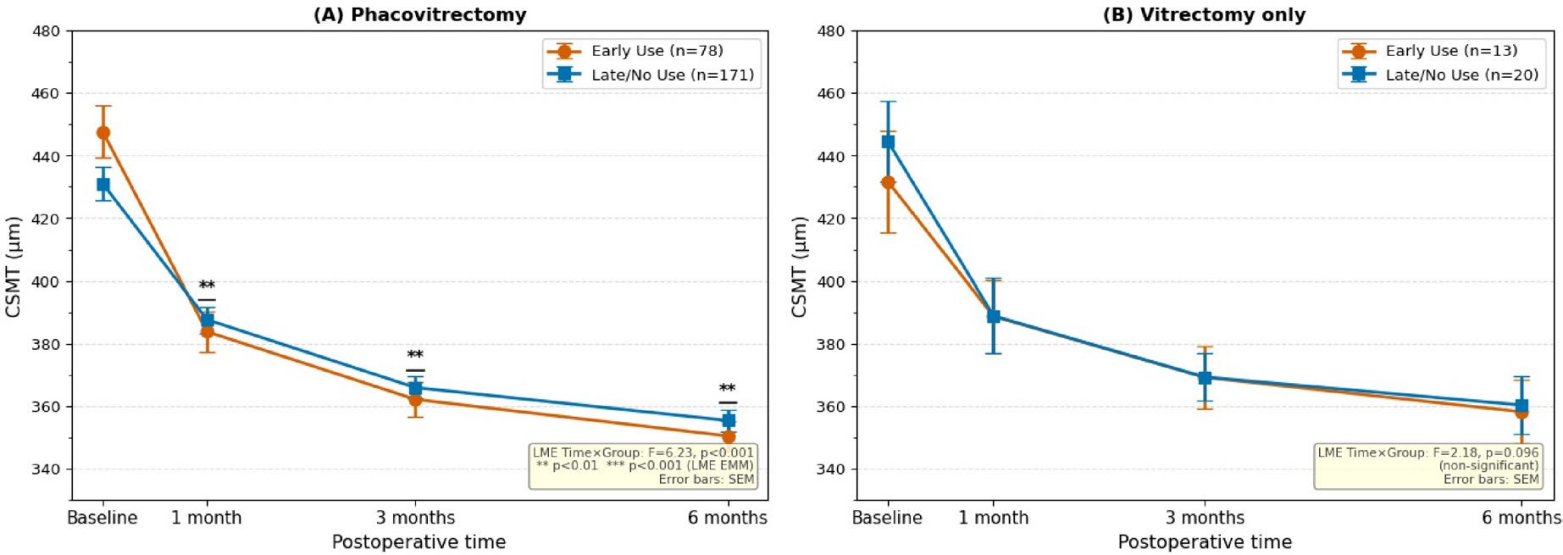
Central subfield macular thickness (CSMT) changes stratified by surgery type. (**A**) In the phacovitrectomy subgroup (Early Use n = 78, Late/No Use n = 171), LME analysis revealed a significant treatment-by-time interaction (F = 6.23, *p* < 0.001). The Early Use Group maintained consistently greater CSMT reduction at 1 month (*P* = 0.003), 3 months (*P* = 0.004), and 6 months (*P* = 0.003). (**B**) In the vitrectomy-only subgroup (Early Use n = 13, Late/No Use n = 20), no significant treatment-by-time interaction was observed (F = 2.18, *P* = 0.096), and no significant between-group differences were detected at any time point. Significance markers indicate LME pairwise comparisons of estimated marginal means. Error bars represent standard error of the mean. Orange circles, Early Use Group; Blue squares, Late/No Use Group.

*Subgroup Analysis: Phacovitrectomy vs. Vitrectomy:* We analyzed the effects of topical bromfenac on CSMT separately for patients undergoing phacovitrectomy and those undergoing vitrectomy alone (Fig. 3). In patients who underwent phacovitrectomy, the difference in CSMT reduction according to the application of topical bromfenac was statistically significant (*P* = 0.006). In contrast, for patients who underwent vitrectomy alone, no significant difference was observed (*P* = 0.981).

## Discussion

The inner blood-retinal barrier (BRB) is maintained by tight junctions between retinal vascular endothelial cells, preventing fluid leakage into the retina. When the BRB is disrupted postoperatively, increased vascular permeability leads to fluid accumulation and CME. Following cataract surgery in particular, this transient BRB breakdown—termed Irvine–Gass syndrome—is largely driven by prostaglandin release. Topical NSAIDs block prostaglandin synthesis and have been established as the preferred treatment for pseudophakic CME, demonstrating superiority over topical steroids for this indication.

In eyes with ERM, the macula is thickened primarily due to mechanical traction exerted by the fibrocellular membrane on the inner retinal surface, though BRB disruption also contributes^2,6^. OCT studies have shown that as ERM severity increases, inner retinal layers including the ganglion cell complex (GCC) and retinal nerve fiber layer (RNFL) become progressively thickened due to the tractional forces applied to the inner retina^8,9^. Following surgical ERM removal, relief of this traction leads to progressive thinning of the inner retinal layers, which correlates with visual acuity improvement^8,9^. This establishes that the natural biological trajectory after ERM surgery is one of progressive inner retinal thinning as traction is relieved — not thickening. Since ERM removal relieves traction and naturally leads to progressive macular thinning, any postoperative macular thickening is more reasonably attributed to increased vascular permeability than to a healing process. In the present study, the significantly greater CSMT in the Late/No Use Group at 1 month, compared with the Early Use Group, might reflect this prostaglandin-mediated vascular response. By inhibiting prostaglandin synthesis during the first 4 postoperative weeks, topical bromfenac suppresses this early vascular permeability component, resulting in the greater and more durable CSMT reduction observed in the Early Use Group.

To the best of our knowledge, two randomized clinical trials have determined whether topical NSAID have an impact on macular thickness after ERM surgery^12,13^.

Schoenberger et al. first studied the efficacy of topical NSAID; however, the results were limited owing to the small number of patients who mainly performed vitrectomy in the cohort (32 eyes)^13^. Mandelcorn et al.^12^ implemented a randomized clinical trial that included 80 patients who underwent ERM surgery and were divided into three groups: topical nepafenac, intravitreal triamcinolone injection, and placebo. They did not find any efficacy of topical nepafenac in postoperative care after ERM removal^12^. Contrary to Mandelcorn et al.’s results^12^, our study showed that postoperative topical NSAID could significantly influence macular thickness changes. We believe the reason for this discrepancy lies in the proportion of phacovitrectomies.

The proportion of patients who underwent phacovitrectomy was only 11.2% (9 eyes) in the study by Mandelcorn et al.^12^, while that was 88.3% (248 eyes) in our study. The results of Mandelcorn et al. and our study imply that combined cataract surgery might be a key factor in the effectiveness of topical NSAIDs. Figure 3 demonstrates that topical NSAIDs were not effective after vitrectomy alone, but were effective after phacovitrectomy, consistent with this hypothesis. This aligns with our previous report comparing CME incidence between phacovitrectomy and vitrectomy, which concluded that CME was triggered not by intraocular surgery itself, but lens removal. The crystalline lens, which is thick and heavy, is replaced by a thin, lighter intraocular lens, a change that induces ciliary body and iris remodeling in which prostaglandin plays important roles^18^.

Our results demonstrate that CSMT decreased most rapidly during the first four postoperative weeks in both groups (Fig. 1). Importantly, macular thickness decreased significantly more in the Early Use Group, and this difference persisted throughout the 6-month follow-up period. Confirming the Mann–Whitney test findings, LME analysis revealed a significant treatment-by-time interaction for CSMT in the full cohort (F = 4.62, *P* = 0.003) and more prominently in the phacovitrectomy subgroup (F = 6.23, *p* < 0.001). Estimated marginal means showed no significant between-group difference at baseline (*P* = 0.164), confirming the comparability of the two groups, but a consistent difference of approximately 14–15 μm emerged at 1 month (*P* = 0.006) and was maintained at 3 months (*P* = 0.004) and 6 months (*P* = 0.003). These findings indicate that the first 4 postoperative weeks represent a critical window during which bromfenac suppresses prostaglandin-mediated macular thickening, establishing an anatomical advantage that persists long after treatment cessation.

LME analysis of logMAR BCVA revealed a significant treatment-by-time interaction in the full cohort (F = 2.68, *P* = 0.046), demonstrating that the two groups followed different trajectories of visual recovery. Early bromfenac use was associated with significantly faster visual acuity recovery at 1 month (EMM difference − 0.054 logMAR, *P* = 0.016), consistent with the earlier macular thickness reduction observed in this group. By 3 months the difference was no longer significant (*P* = 0.503), and by 6 months both groups showed equivalent visual acuity (EMM: Early Use 0.213 vs. Late/No Use 0.191, *P* = 0.305), demonstrating convergence of visual outcomes at the end of follow-up. The finding that early bromfenac accelerates visual recovery without affecting the final visual outcome underscores the principle that long-term visual acuity after ERM surgery is ultimately determined not by the early postoperative macular edema, but by the integrity of microstructural elements such as the ellipsoid zone, ectopic inner foveal layers, and foveal photoreceptor density^8,9^. The convergence of visual acuity at 6 months, despite persistently thinner CSMT in the Early Use Group throughout follow-up, suggests that the anatomical benefit of early bromfenac use does not translate into a sustained functional advantage over a 6-month period. This finding is consistent with the view that the prostaglandin-mediated vascular response in the early postoperative period influences short-term macular edema but does not independently determine the long-term microstructural recovery of the foveal photoreceptors.

The cellular consequences of ILM removal are also relevant to interpreting the long-term microstructural recovery after ERM surgery. The ILM serves as the basement membrane formed by Müller cell footplates, and its removal directly disrupts these glial endfeet. A recent comprehensive review by Sadeghi et al.^19^ reported that retinal nerve fiber layer (RNFL) damage following ILM peeling is attributed to ganglion cell degeneration and axonal transport alteration, while the dissociated optic nerve fiber layer (DONFL) appearance is a direct consequence of Müller cell damage. Furthermore, ILM removal has an overall thinning effect on the retina, reflecting loss of Müller cell structural support and progressive inner retinal remodeling. These cellular changes underscore that ILM peeling is not a biologically neutral procedure, and that long-term microstructural recovery after ERM surgery involves complex glial and neuronal remodeling processes. This further supports the interpretation that postoperative visual outcomes are ultimately governed by inner retinal microstructural integrity rather than macular thickness alone.

Several limitations of this study merit consideration. First, the retrospective design means that postoperative regimens and follow-up schedules were not perfectly standardized across surgeons, and the criteria for initiating bromfenac in the Late/No Use Group were not uniform. In the Early Use Group, early bromfenac prescription was not based on specific clinical indications documented in individual patient records, but is believed to have reflected a change in the routine postoperative prescription protocol among individual surgeons during the study period. Second, no fluorescein angiography or OCT-A was performed, limiting direct assessment of vascular permeability changes. Third, while ERM structural staging (Govetto et al.) was assessed and showed no significant difference in stage distribution between groups (*P* = 0.832), the relatively small number of stage 4 eyes limits subgroup analysis by ERM severity; future studies with larger samples may clarify whether bromfenac efficacy varies by ERM stage. Fourth, the relatively small vitrectomy-only subgroup limits the statistical power to draw definitive conclusions about the lack of bromfenac effect in that group. Fifth, a qualitative bias assessment informed by the ROBINS-I framework identified the following potential sources of bias: confounding bias due to the non-randomized assignment of bromfenac timing; selection bias inherent to the retrospective multi-surgeon design; and possible classification bias in the Late/No Use Group, as 14.1% of patients ultimately received bromfenac after week 4. To assess the robustness of the primary findings, a sensitivity analysis was conducted excluding these 27 eyes; the treatment-by-time interaction for CSMT remained statistically significant, supporting the stability of the main results. Finally, the 6-month follow-up period may be insufficient to determine permanent structural outcomes, and confirmation through larger-scale prospective studies with longer follow-up is warranted.

In conclusion, early postoperative topical bromfenac within 4 weeks of phacovitrectomy for ERM produces a significantly greater reduction in macular thickness that is durably maintained for up to 6 months, along with accelerated visual acuity recovery at 1 month. These findings raise the hypothesis that prostaglandin-mediated vascular permeability in the early postoperative period may influence long-term macular remodeling; however, this remains speculative, as direct evidence of permeability changes was not assessed in this study.

## Data Availability

The datasets generated and/or analyzed during the current study are available from the corresponding author on reasonable request.
